# Characterization of novel small non-coding RNAs and their modifications in bladder cancer using an updated small RNA-seq workflow

**DOI:** 10.3389/fmolb.2022.887686

**Published:** 2022-07-18

**Authors:** Zhangli Su, Ida Monshaugen, Arne Klungland, Rune Ougland, Anindya Dutta

**Affiliations:** ^1^ Department of Genetics, University of Alabama at Birmingham, Birmingham, AL, United States; ^2^ Department of Biochemistry and Molecular Genetics, School of Medicine, University of Virginia, Charlottesville, VA, United States; ^3^ Department of Microbiology, Oslo University Hospital Rikshospitalet, Oslo, Norway; ^4^ Department of Molecular Medicine, Institute of Basic Medical Sciences, University of Oslo, Oslo, Norway; ^5^ Department of Biosciences, Faculty of Mathematics and Natural Sciences, University of Oslo, Oslo, Norway; ^6^ Department of Surgery, Baerum Hospital Vestre Viken Hospital Trust, Gjettum, Norway

**Keywords:** bladder cancer, non-coding RNA, small RNA, RNA modification, tRNA-derived fragment, rRNA-derived fragment, YRNA-derived fragment

## Abstract

**Background:** Bladder cancer (BLCA) is one of the most common cancer types worldwide. The disease is responsible for about 200,000 deaths annually, thus improved diagnostics and therapy is needed. A large body of evidence reveal that small RNAs of less than 40 nucleotides may act as tumor suppressors, oncogenes, and disease biomarkers, with a major focus on microRNAs. However, the role of other families of small RNAs is not yet deciphered. Recent results suggest that small RNAs and their modification status, play a role in BLCA development and are promising biomarkers due to their high abundance in the exomes and body fluids (including urine). Moreover, free modified nucleosides have been detected at elevated levels from the urine of BLCA patients. A genome-wide view of small RNAs, and their modifications, will help pinpoint the molecules that could be used as biomarker or has important biology in BLCA development.

**Methods:** BLCA tumor tissue specimens were obtained from 12 patients undergoing transurethral resection of non-muscle invasive papillary urothelial carcinomas. Genome-wide profiling of small RNAs less than 40 bases long was performed by a modified protocol with TGIRT (thermostable group II reverse transcriptase) to identify novel small RNAs and their modification status.

**Results:** Comprehensive analysis identified not only microRNAs. Intriguingly, 57 ± 15% (mean ± S.D.) of sequencing reads mapped to non-microRNA-small RNAs including tRNA-derived fragments (tRFs), ribosomal RNA-derived fragments (rRFs) and YRNA-derived fragments (YRFs). Misincorporation (mismatch) sites identified potential base modification positions on the small RNAs, especially on tRFs, corresponding to m^1^A (N^1^-methyladenosine), m^1^G (N^1^-methylguanosine) and m^2^_2_G (N^2^, N^2^-dimethylguanosine). We also detected mismatch sites on rRFs corresponding to known modifications on 28 and 18S rRNA.

**Conclusion:** We found abundant non-microRNA-small RNAs in BLCA tumor samples. Small RNAs, especially tRFs and rRFs, contain modifications that can be captured as mismatch by TGIRT sequencing. Both the modifications and the non-microRNA-small RNAs should be explored as a biomarker for BLCA detection or follow-up.

## Introduction

Bladder cancer (BLCA) is the sixth most common cancer worldwide with high morbidity and mortality rates. With 550,000 annual new incidents and 200,000 deaths, BLCA poses a significant disease burden globally (Bray et al., 2018). About 75% of incidents present as non-muscle invasive (NMIBC), consisting of a heterogeneous population of tumors (Kirkali et al., 2005; Burger et al., 2013). Currently there is no routine screening for NMIBC or BLCA in general. Patients with NMIBC usually display urinary tract symptoms i.e., hematuria, pain or frequent urination, and is then subject to cystoscopy as the first step in the diagnostic process. If the initial workup reveals a tumor, the affected individual often undergoes surgery. In addition, patients may receive radiation therapy, chemotherapy, immunotherapy and targeted therapy. Despite a 70–80% recurrence rate, NMIBC has a favorable prognosis and a 5-year survival rate greater than 85% (van Rhijn et al., 2009). However, up to 30% of NMIBC cases progress into more advanced stages with less favorable prognosis, and 5-year survival rate drops to about 5% for metastatic disease (Schrier et al., 2004; Sanli et al., 2017; Boegemann and Krabbe, 2020). This lifelong menace necessitates an exhaustive post-operative control scheme burdening both patients and healthcare systems. In fact, BLCA is in the top tier of the most expensive cancer type to treat, both when considering cost per patient and lifetime cost, in addition to the invaluable expense of life quality reduction (James and Gore, 2013). Thus, urgent improvement of diagnostics and follow-up is required. Despite tremendous effort, the development of sensitive biomarkers and non-invasive methods for cost-effective diagnostics and surveillance of patients remains a challenge. However, the family of small non-coding RNAs and their modifications, appear as a promising addition to the future clinical toolbox.

Non-coding RNAs (ncRNAs), including both long non-coding RNAs (lncRNAs) and small RNAs (sRNAs), have gained much attention lately for their key role as mediators of gene expression in cancer (Slack and Chinnaiyan, 2019). They are considered well-suited as therapeutic targets or agents due to their small size and chemical properties, which allow them to cross tissue barriers and reach tumor cell interior better than macromolecular antibody drugs (Li et al., 2020). In particular, the primary focus of sRNA research in BLCA has been directed towards microRNAs (miRNAs). Extensive RNA sequencing by The Cancer Genome Atlas reported epigenetic regulation of ncRNA, especially miRNAs, in BLCA (Yoshino et al., 2013; Cancer Genome Atlas Research, 2014). In recent years, dysregulated expression of hundreds of miRNAs have been reported in BLCA by large-scale analysis from close to 20 research groups (Lee et al., 2016). Functional studies suggest that miRNAs are involved in different aspects of BLCA development and progression (Li et al., 2011; Morais et al., 2014; Wang et al., 2015). Moreover, miRNAs dysregulated in tumor tissue can also be detected in biological fluids such as serum and urine, suggesting their potential usage as non-invasive diagnostic or prognostic tools (Yun et al., 2012; Armstrong et al., 2015; Fang et al., 2016; Borkowska et al., 2019; Yin et al., 2019).

Besides microRNAs, other emerging small RNAs are detected at high abundance thanks to the technological advances in next-generational sequencing. For example, tRNA-derived fragments (tRFs) have gained attention as their diverse biological functions are being discovered (Magee and Rigoutsos, 2020). These sRNAs have high promise due to their biological functions in different diseases and their high abundance in bodily fluids as recently reviewed (Su et al., 2020b). So far only a few reports focused on tRFs or other non-microRNA-small RNAs in BLCA. tRF expression showed context-dependent association with mRNA expression across 32 cancer types in TCGA, highlighting differences in tRF-mRNA connection by sex in bladder cancer (Telonis et al., 2019). Furthermore, analysis of TCGA data found association between elevated level of a specific tRF (5′-tRF-LysCTT) and early progression and poor outcome in BLCA (Papadimitriou et al., 2020), calling for further investigation of tRF functions in BLCA. In addition to tRFs, other small RNAs such as ribosomal RNA-derived fragments (rRFs) and Y RNA-derived fragments (YRFs) have also been reported in humans but not investigated as much. Important to note, TCGA small RNA-seq data was collected focusing on microRNAs (∼22 nucleotides long) and has a strict size cut-off of 30 nucleotides, losing potential information on RNAs longer than this size range.

Moreover, sRNAs harbor a range of chemical modifications providing a second layer of biological information (Zhang et al., 2016; Li et al., 2021). This modification information is often missing and even leads to under-representation of modification-containing sRNAs during conventional small RNA-seq library preparation (Shi et al., 2021). Enzyme-assisted library preparation improves the cloning of modification-containing sRNAs, and suggests that their abundance was formerly far under-appreciated. Altogether, there is a great need to understand the relative abundance of non-microRNA-small RNAs and modification status on different small RNAs, both of which have not been comprehensively profiled in BLCA samples.

We aimed to establish a workflow that can profile both microRNA (miRs) and non-miR small RNAs in a genome-wide fashion that can be applied to patient samples. Small RNA-seq has been a powerful method for high-throughput profiling and sequence-level information that is important for base-level analysis. However, regular small RNA-seq protocol is known to suffer from the stalling of the reverse transcriptase at sites containing modifications that disrupt Watson-Crick base-pairing, including but not limited to m^1^A (N^1^-methyladenosine), m^1^G (N^1^-methylguanosine), and m_2_^2^G (N^2^, N^2^-dimethylguanosine) (Behrens et al., 2021; Shi et al., 2021). Recently we showed TGIRT (thermostable group II intron reverse transcriptase) can be used in small RNA-seq to overcome under-cloning of m^1^A-containing RNAs during regular small RNA-seq protocol, and further be used to identify the modification base position via mismatch (Su et al., 2022). The under-representation of m^1^A-containing small RNAs and loss of quantitative mismatch ratio by a commonly used M-MuLV reverse transcriptase (ProtoScriptII) indicates the regular small RNA-seq pipeline is biased against m^1^A-modified small RNAs. Intrigued by this result, we wondered whether TGIRT can also overcome and capture the other RNA modifications that disrupt Watson-Crick base-pairing. In addition to A-type mismatch, we noticed TGIRT also produced more G-type mismatch than ProtoScriptII from the same HEK293T RNAs (Supplementary Figure S1A), suggesting TGIRT can potentially capture modifications on guanosine as well. Here we report a comprehensive profiling of small RNAs and their modification status in BLCA patient samples by this modified small RNA-seq pipeline. From 12 tumor samples, we identified non-microRNA-small RNA reads that are comparable in abundance to microRNAs. These non-microRNA-small RNAs include tRNA-fragments, rRNA-fragments, Y-RNA-fragments, snoRNA-fragments and more. Their length distribution and cleavage patterns were distinctly different. RNA modification as indicated by TGIRT mismatch pattern was mostly found on tRFs over other small RNA types. Mismatch sites on specific tRFs correspond to known m^1^A, m^1^G and m_2_^2^G annotations on mature tRNAs, suggest a large proportion of tRFs harbor these modifications. Furthermore, mismatch sites were also identified on rRFs at known modification positions on 28 and 18S rRNAs. This analysis confirms the high potential of using TGIRT to enable modification-friendly profiling of small RNAs in clinical samples.

## Materials and methods

### Human subject and sample collection

Patients diagnosed and treated for papillary urothelial NMIBC at the Vestre Viken Hospital Trust hospitals were enrolled in the study. Cold cup biopsies were harvested prior to surgical resection of the tumor, and the specimens were kept on −20°C in RNAlater preservation solution (Ambion #AM7020) until preparation and further analyses.

Anonymized collective patient information of the 12 samples used is listed in Table 1.

**TABLE1:** NMIBC patient information.

Patient #	Sex	Age range	Primary or recidive
Patient #1	Female	40-49	Primary
Patient #2	Male	70-79	Primary
Patient #3	Male	70-79	Primary
Patient #4	Male	60-69	Primary
Patient #5	Male	70-79	Primary
Patient #6	Male	>80	Primary
Patient #7	Male	60-69	Primary
Patient #8	Male	50-59	Primary
Patient #9	Male	40-49	Primary
Patient #10	Male	50-59	Primary
Patient #11	Male	>80	Primary
Patient #12	Female	70-79	Primary

### RNA extraction

Purification of total RNA was done using the RNAzol RT reagent (MRC Inc. #RN190). Subsequently RNA quality was determined using RNA ScreenTape on TapeStation (Agilent Tech. #5067-5576) or Agilent RNA 6000 Pico Kit on Bioanalyzer (Agilent Tech. #5067-1513).

### Small RNA library preparation by TGIRT and sequencing

Small RNA-seq library preparation was performed as previously reported (Su et al., 2019; Su et al., 2020a) using NEBNext Small RNA Library Prep Set for Illumina (NEB #7330) with changes to use TGIRT for cDNA synthesis. TGIRT condition is based on m^1^A mapping on polyA-enriched RNAs by TGIRT-seq (Li et al., 2017) with the modifications described below. Total RNAs of 0.3–1 μg were ligated with the corresponding 3′ and 5’ adaptor within the NEBNext kit. Ligated RNAs were converted to cDNA by 1 μL TGIRT-III enzyme (InGex #TGIRT50) per reaction at 60°C for 15 min. TGIRT reaction was carried out in buffer (50 mM Tris, pH 8.3, 75 mM KCl, 3 mM MgCl_2_, 1 mM dNTP, 10 mM DTT) with addition of 1 μL RNase Inhibitor. The reaction is stopped by addition of 250 mM (final concentration) NaOH at 95°C for 3 min and 65°C for 15 min. Same amount of HCl was added to neutralize the reaction after the reaction cools down. The cDNA is further purified by QIAquick Nucleotide Removal Kit (Qiagen #28304) or ZYMO oligo clean and concentrator kit (ZYMO #D4060) or Dynabeads MyOne Silane (Thermo Fisher #37002D). cDNA is amplified by 15–16 cycles of PCR with indexed NEBNext primers (NEB #E6609). The individual amplified libraries were purified with ZYMO DNA Clean and Concentrator Kit (ZYMO #D4033) and run on 8% TBE polyacrylamide Novex gel (Thermo Fisher #EC6215). The position corresponding to RNA insert of 15–40 nucleotides long was cut out from the gel and purified *via* crush-and-soak method. Care was taken to cut the region longer than primer dimer and shorter than full-length tRNA. Gel-recovered eluate was purified and concentrated by ethanol precipitation according to NEB kit instruction. Final libraries were quantified by Qubit fluorometer and pooled for sequencing on Illumina sequencer.

HEK293T small RNA-seq data by ProtoScriptII and TGIRT can be accessed from GEO: GSE171040 (GSM5217188 and GSM5217193 for ProtoScriptII; GSM5217184 and GSM5217186 for TGIRT).

### General mapping strategy for small RNA TGIRT-seq data analysis

Small RNA-seq data was analyzed similarly as before (Su et al., 2019; Su et al., 2020a). Briefly, cutadapt v1.15 (Martin, 2011) was used to trim 3′ adaptor sequence and discard trimmed read length shorter than 15 nt. To avoid mis-annotation of 5′ NEBNext adaptor sequence to hsa-miR-3168, reads containing 5′ adaptor sequence were discarded with cutadapt. In general, each library has 2–10 million mapped reads. Unitas v1.7.3 (Gebert et al., 2017) with SeqMap v1.0.13 (Jiang and Wong, 2008) was used to map small RNAs. Priority of mapping was given to first map the reads to miRBase Release 22 (Kozomara et al., 2019) human sequence. The remaining reads were mapped to other small RNA sequences including genomic tRNA database (Chan and Lowe, 2016) and Ensembl Release 97. Additional rRNA and YRNA reference sequences were used for rRF and YRF mapping: 18S (NR_145820.1), 5S (NR_023363.1), 28S (NR_003287.4) and 5.8S (NR_145821.1); RNY1 (NR_004391.1), RNY3 (NR_004392.1), RNY4 (NR_004393.1) and RNY5 (NR_001571.2). miRNA mapping was done allowing 2 non-templated 3’ nucleotides addition and 1 internal mismatch. Other nmsRNA mapping was done allowing 1 mismatch and 0 insertion/deletion, unless otherwise specified (for example Supplementary Figure S1). When a given read is mapped to multiple reference RNAs (multi-mapping), fractionated count was calculated assuming even distribution among all possible references. For all the analysis, reads per million (RPM) was calculated to adjust for total mapped reads in each library.

### Mismatch calculation for small RNA TGIRT-seq data analysis

After initial mapping as above, tRF/rRF/YRF reads were re-mapped to only the corresponding reference RNAs with unitas v1.7.3 (Gebert et al., 2017) allowing 1 mismatch and 0 insertion/deletion. The mapping start/end position and mismatch position were recorded for each read. The reads were aggregated onto the whole length of reference RNAs into a coverage plot to facilitate visualization, with *X* axis representing each nucleotide position of the reference RNA and *Y* axis representing the abundance of all reads that covered that specific position. Mismatch index (on a scale of 0–100%) was calculated for each position by taking the reads with mismatch at that position and divided by total reads that covered that specific position. Mismatch index was visualized by color on the coverage plot (red means higher mismatch). For tRFs (Figure 4), coverage was aggregated by tRNA amino acid groups.

### Coverage plot on secondary structure of YRNA-derived fragments

YRNA secondary structures were retrieved from RNAcentral v19 based on Rfam (RF00019). The dot-bracket notation was used to generate secondary structure plot by StructureEditor v6.1. To color each base based on the relative abundance, coverage of each base is normalized to the highest coverage for that YRNA.

## Results

### An updated small RNA-seq workflow for modification-friendly global analysis

**FIGURE 1:**
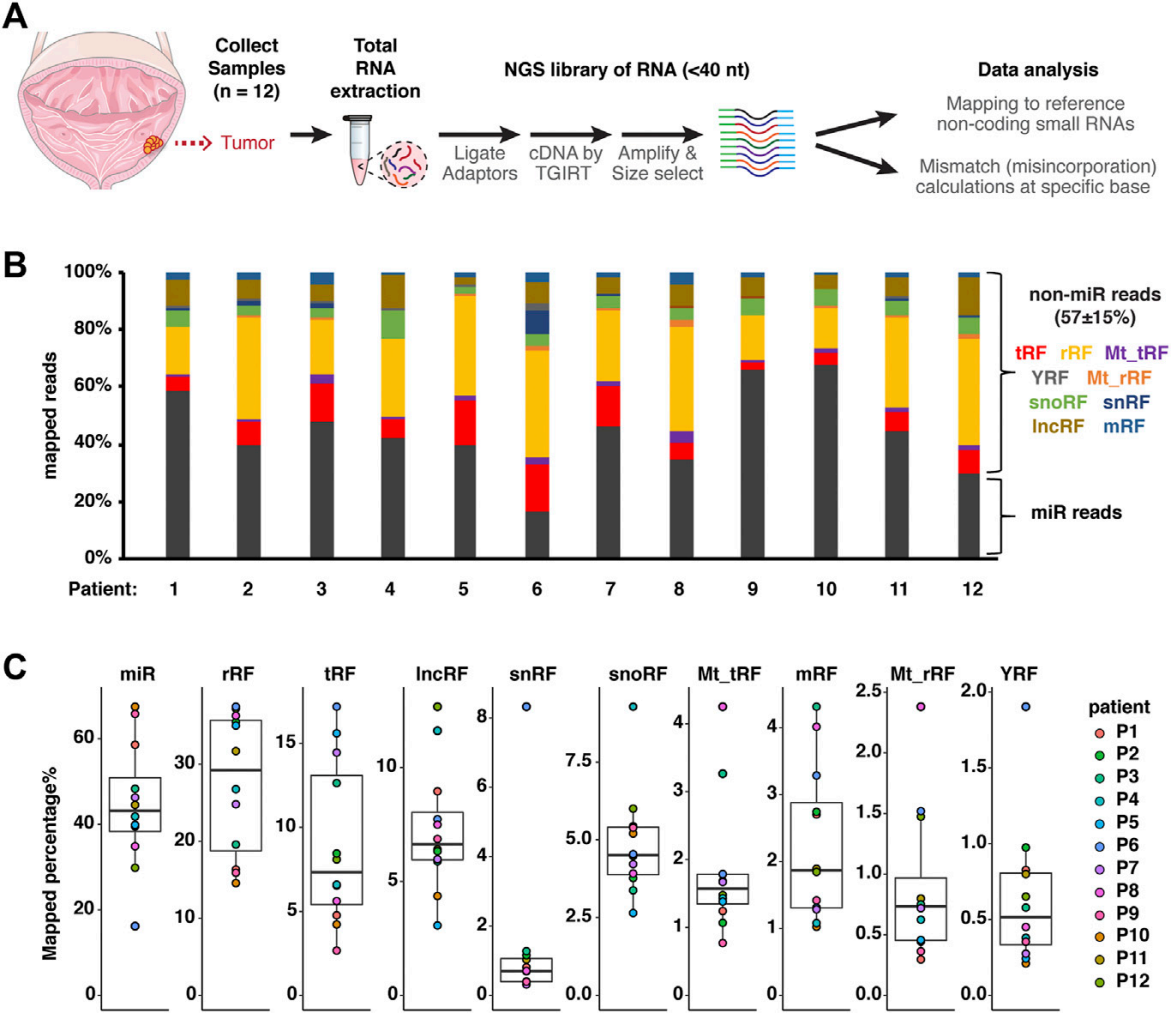
An updated small RNA-seq workflow for modification-friendly global analysis. **(A)** Scheme of collecting tumor samples from 12 NMIBC patients. Small RNA-sequencing libraries by TGIRT were prepared from total RNAs to profile relative abundance and potential RNA modifications (based on mismatch/misincorporation) for small RNAs less than 40 bases long. **(B)** Overall distribution of total mapped reads between microRNAs (dark grey) and non-microRNA small RNAs (nmsRNAs), including rRFs (yellow), tRFs (red) and more. **(C)** Distribution of mapped percentage for each sub-group of small RNAs shown as box-whisker plot. Box plot center represents median value, bounds represent upper and lower quartile, whiskers represent 1.5* interquartile range from the bounds.

We collected 12 NMIBC tumor samples (patient information summarized in Table 1) to test the updated small RNA-seq workflow (Figure 1A). High-quality total RNAs were used as input and first ligated with 3′ adaptor and 5’ adaptor. Ligated RNA was converted into cDNA by reverse transcriptase TGIRT, which has been shown to produce more mismatch (misincorporation) than hard-stop products at m^1^A modification sites than other reverse transcriptases (Li et al., 2017; Su et al., 2022). When sequencing HEK293T RNAs, we noticed TGIRT protocol is better at capturing non-microRNA-small RNAs than the regular ProtoScriptII protocol (Supplementary Figure S1A). The higher G-type mismatch from the same HEK293T RNAs by TGIRT (Supplementary Figure S1B), suggests TGIRT might detect certain modifications on guanosine as well. To be noted, base modifications that disrupt base pairing such as m^1^A, m^1^G, m_2_^2^G are more prone to produce RT-induced mismatch, while other RNA modifications including m^6^A and pseudouridine are less affected. Only the fully ligated and converted cDNAs can be further amplified by the next PCR amplification. Lastly, the PCR products are size selected experimentally to enrich for small RNAs of size less than 40 nucleotides long. To avoid ambiguous mapping of very short sequences, we only mapped reads that are at least 15 nucleotides long. Each clean read was first mapped to human microRNA sequences, and the remaining reads were then mapped to other reference sequences to identify non-microRNA-small RNAs (nmsRNA) (Supplementary Figure S1C). The largest increase in mapping of nmsRNAs (of 25–150%) was seen specifically with tRFs compared to other nmsRNAs (Supplementary Figure S1D). In contrast allowing indels of 1 nt did not increase mapping numbers (Supplementary Figure S1E). Overall we found a very high percentage of nmsRNA reads in these libraries, constituting 42–72% of all mapped reads (Figure 1B). The most abundant nmsRNAs include tRNA-derived fragments (tRFs), ribosomal RNA-derived fragments (rRFs), mitochondrial tRFs, mitochondrial rRFs, Y-RNA-derived fragments (YRFs), small nucleolar RNA-derived fragments (snoRFs), small nuclear RNA-derived fragments (snRFs), lncRNA-derived fragments (lncRFs) and protein-coding mRNA-derived fragments (mRFs). Among these, the four most abundant groups are rRFs, tRFs, snoRFs and lncRFs (Figure 1B). It was striking that in some patients (patients 2, 5, 6, 8 and 12), rRF read counts were nearly equal to or more than microRNAs. Interestingly, relative read distribution among different small RNA sub-groups was quite variable for different patients (Figure 1C).

### microRNAs and nmsRNAs (non-microRNA-small RNAs) show distinct size distribution

**FIGURE 2:**
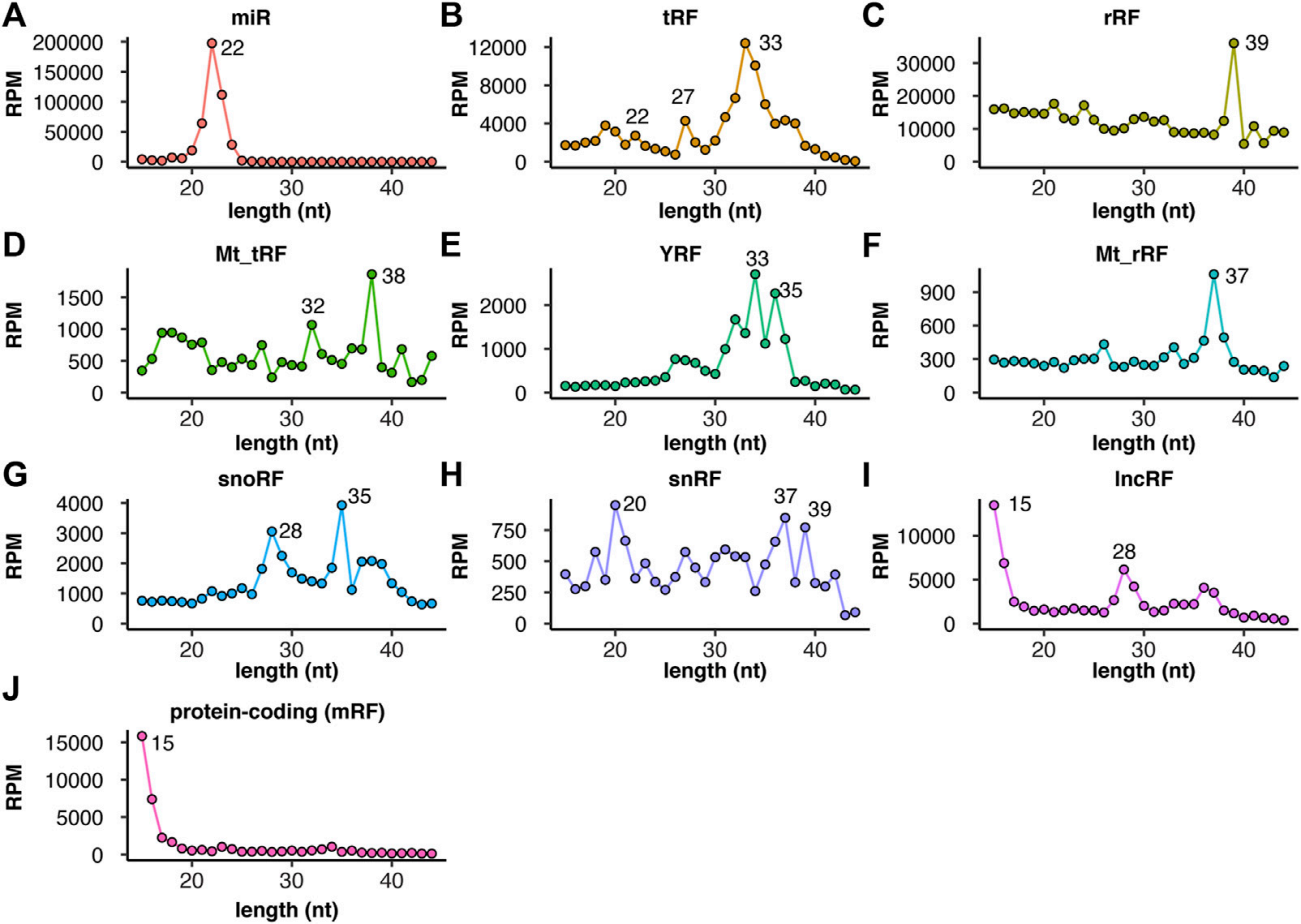
microRNAs and non-microRNA small RNAs show distinct size distribution. Size distribution (*X*-axis in nucleotides, *Y*-axis in reads per million mapped reads) of each subtype of small RNAs including **(A)** microRNAs, **(B)** tRFs, **(C)** rRFs, **(D)** mitochondrial tRFs, **(E)** YRFs, **(F)** mitochondrial rRFs, **(G)** snoRFs, **(H)** snRFs, **(I)** lncRFs and **(J)** mRFs. Major peaks in length are labeled for each small RNA subtype. RPM values are averaged from 12 samples (individual samples plotted in Supplementary Figure S2).

To further understand the characteristics of the nmsRNAs, we checked the length distribution of each subtype. As expected, microRNAs have a specific size of 22 nucleotides (average from 12 samples shown in Figure 2A, individual patient samples shown in Supplementary Figure S2). Meanwhile, the other small RNAs showed distinct size distributions that were different from microRNAs. For example, tRFs (Figure 2B), rRFs (Figure 2C), mitochondrial tRFs (Figure 2D), YRFs (Figure 2E), mitochondrial rRFs (Figure 2F) and snoRFs (Figure 2G) all have a longer size range than microRNAs. snRFs have a peak at 20 nucleotides and additional peaks at longer size of 37 and 39 nucleotides (Figure 2H). This also suggests these longer nmsRNAs were missed or under-represented if a library was size selected around 22 nucleotides.

Similar to what we found before (Kumar et al., 2014), tRFs display specific peaks in length at 33, 27, 22 and 18 nucleotides (Figure 2B), which will be further discussed in the next section. Intriguingly, mitochondrial tRFs display additional peaks at 38 and 32 nucleotides (Figure 2D). In addition, both genomic and mitochondrial rRFs are represented by a very specific peak (39 and 37 nucleotides) (Figure 2C and Figure 2F). YRFs have peaks of 33, 35 and 31 nucleotides (Figure 2E), whereas snoRFs have peaks of 35 and 28 nucleotides (Figure 2G). On the other hand, lncRFs and mRFs have predominantly shorter reads of 15 nucleotides, (Figures 2I–J) which is the size cut-off for our bioinformatics analysis (we discarded reads shorter than 15 nucleotides to avoid ambiguous mapping). This may suggest more non-specific cleavage on lncRNA and mRNAs than the other RNAs. In general, the pre-dominant size for each small RNA subtype was consistently observed across 12 tumor samples, which shows distinct pattern between different RNA subtypes (Supplementary Figure S2). Below we describe specific nmsRNA subtypes in more details.

### TGIRT-seq detects tRNA halves and tRFs in the microRNA-size range at an abundance comparable to microRNAs

**FIGURE 3:**
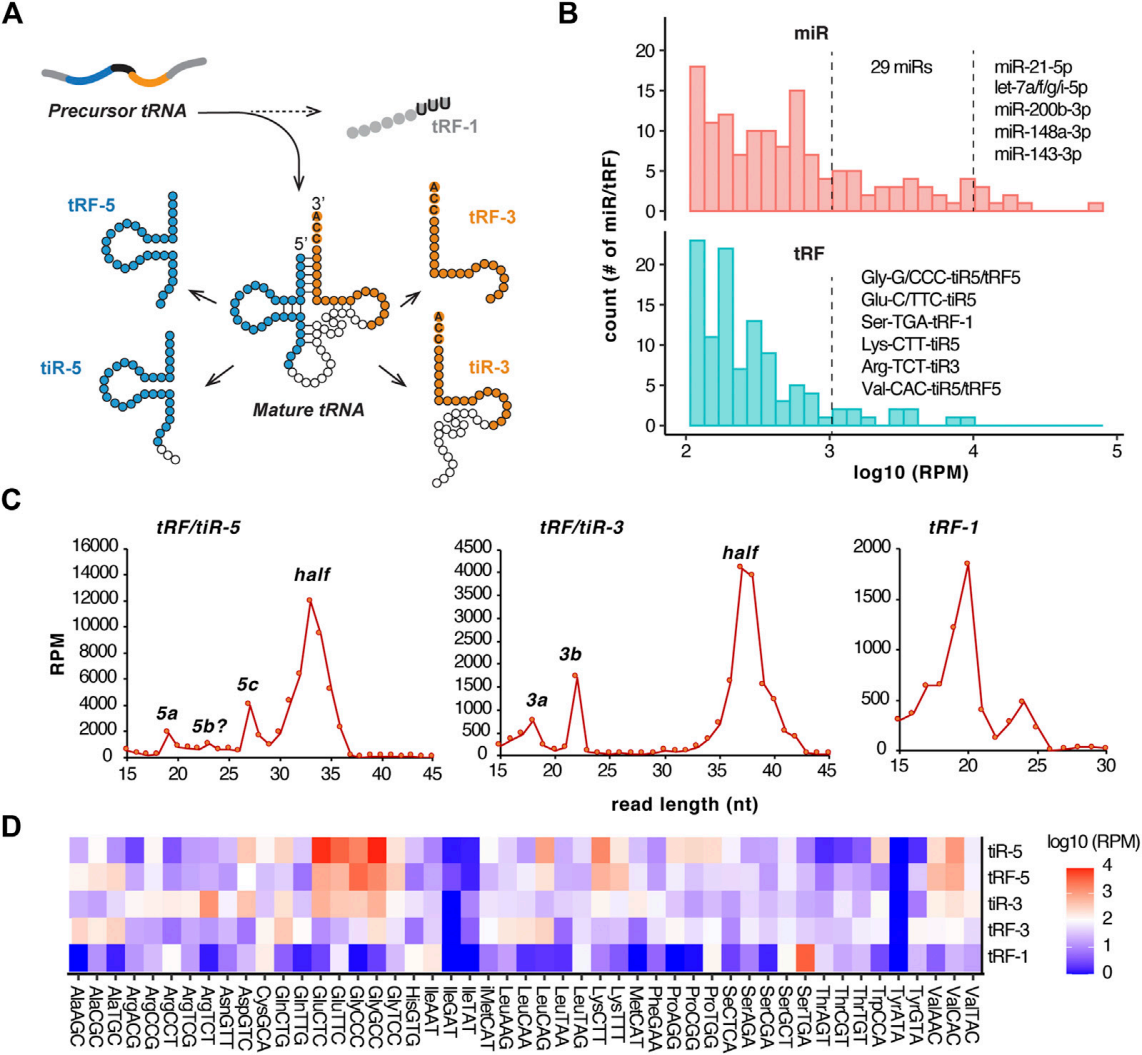
Abundant tRFs show distinct size distribution. **(A)** Major subtypes of tRFs. **(B)** Comparison of relative abundance of microRNAs and tRFs by histogram (*X*-axis: log 10 scale of reads per million). Top expressed miRs and tRFs (RPM >1,000) are labeled. tRF is grouped by each of the five types in **(A)** and further by anticodon. **(C)** Size distribution (*X*-axis in nucleotides, *Y*-axis in reads per million mapped reads) of each tRF subtype. **(D)** tRF abundance shown as a heatmap grouped by tRF types and anticodons. RPM values are averaged from 12 samples (individual samples plotted in Supplementary Figure S3).

tRFs are grouped by their start and end positions on parental tRNAs, including 5′ fragments and 3′ fragments from mature tRNAs and tRF-1s from precursor tRNA trailers (Figure 3A). Both 5′ and 3′ fragments can be further divided into longer fragments (or called tRNA halves, tiRs) and shorter tRFs. Generally, expression levels for microRNAs are higher than that of tRFs (Figure 3B, one-sided Kolmogorov-Smirnov test, p = 1E-5). The most abundant microRNAs detected by TGIRT-seq include miR-21-5p, let-7-5p, miR-200b-3p, miR-148a-3p and miR-143-3p (each more than 10,000 reads per million). When compared with these highly abundant microRNAs, specific tRFs are also expressed at high levels, including 5′ halves/fragments from tRNA^Gly^, tRNA^Glu^, tRNA^Lys^, tRNA^Val^, 3’ half from tRNA^Arg^ and tRF-1 from tRNA^Ser^, all of which exceed 1,000 reads per million averaged from 12 samples (Figure 3B). The abundance (1,000-10,000 RPM) of these five tRFs is comparable to that of 29 unique microRNAs (let-7b-5p, let-7e-5p, miR-100-5p, miR-101-3p, miR-103a-3p, miR-10a-5p, miR-10b-5p, miR-125a-5p, miR-126-3p, miR-148b-3p, miR-151a-3p, miR-191-5p, miR-199a-3p, miR-200a-3p, miR-200c-3p, miR-203a-3p, miR-205-5p, miR-23a-3p, miR-23b-3p, miR-25-3p, miR-26a-5p, miR-26b-5p, miR-27a-3p, miR-27b-3p, miR-30d-5p, miR-378a-3p, miR-92a-3p, miR-98-5p, miR-99b-5p).

Both 5′ and 3′ fragments have a major peak corresponding to the tRNA halves that are cleaved in the anticodon loop, with other minor peaks representing shorter isoforms (average from 12 samples shown in Figure 3C, individual patient samples shown in Supplementary Figure S3). 5′ fragments have dominant size of 32–34 nt (5′ halves), 27 nt (tRF-5c) and 19 nt (tRF-5a). 3′ fragments have dominant size of 37–38 nt (3’ halves), 22 nt (tRF-3b) and 18 nt (tRF-3a). tRF-1s are generally shorter than 25 nt with a major peak at 20 nt (Figure 3C). Again, the size distribution pattern is overall consistent among 12 samples (Supplementary Figure 3).

tRF reads are derived from different tRNA genes (Figure 3D). tRF-1 expression shows the lowest correlation with the other tRF types, with the highest tRF-1 expression from tRNA-Ser-TGA (tRF-1001). The most abundant fragments are tiR-5, tRF-5 and tiR-3 from tRNA-Glu-C/TTC and tRNA-Gly-C/GCC. tRF-3s have highest expression from tRNA^Gln^, tRNA^Leu^ and tRNA^Ala^.

### TGIRT-seq captures mismatch at specific positions corresponding to RNA m^1^G, m_2_^2^G and m^1^A modification sites

**FIGURE 4:**
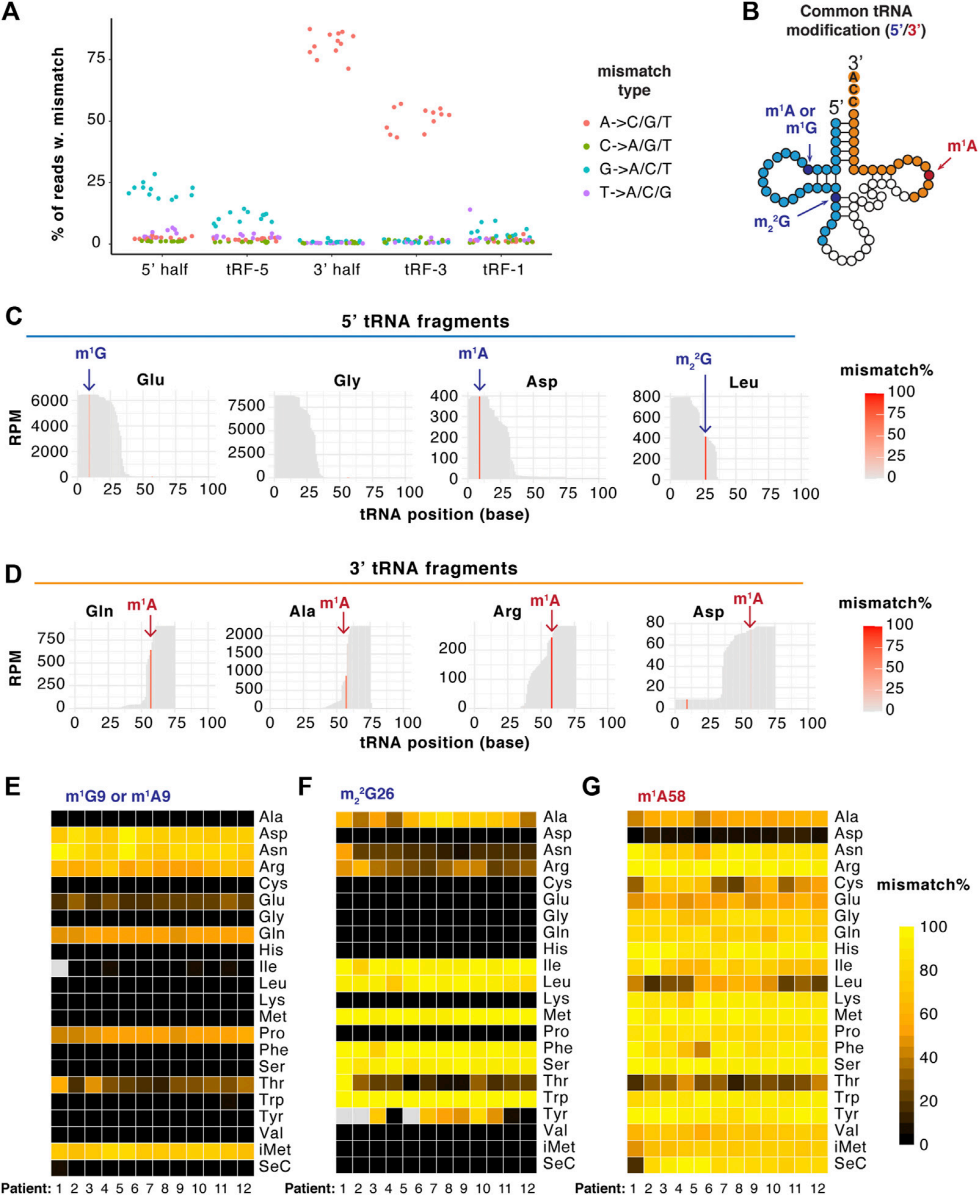
TGIRT-seq captures mismatch at specific positions corresponding to RNA modification sites. **(A)** A- and G-type mismatch is abundantly detected in tRFs by TGIRT-seq. In particular, A-type mismatch is enriched in 3′ fragments while G-type mismatch is enriched in 5′ fragments. Each dot represents one patient sample (*n* = 12), separated by mismatch types (by color) and tRF types (*X*-axis). *Y* axis represents the percentage of reads that contain specific type of mismatch. **(B)** Scheme of known common tRNA modifications that are detected on tRFs by TGIRT. **(C,D)** Example coverage plot of 5’ **(C)** and 3’ **(D)** tRNA fragments with mismatch positions highlighted at each position (patient #1 shown as example). **(E–G)** Heatmap of tRF mismatch index (percentage) at specific positions representing m^1^G/A9 **(E)**, m_2_^2^G26 **(F)** and m^1^A58 **(G)**. All tRF reads are combined and clustered on the length of parental tRNAs. Each column represents one tumor sample (*n* = 12). Grey squares represent no read coverage at that site.

Allowing one nucleotide mismatch increased tRF mapping (Supplementary Figure S1), suggesting tRFs likely bear mismatch-inducing RNA modifications. We checked what type of mismatch was captured in the TGIRT library and found around 15% of tRF reads contain A- > C/G/T mismatch and 15% contain G- > A/C/T mismatch, both of which are much higher than the percentage seen in microRNA reads (Supplementary Figure S4). This is consistent with the fact that tRNAs bear an array of RNA modifications, including modified adenosines and guanosines (Clark et al., 2016; Behrens et al., 2021). Specifically, the G-type mismatch mainly happens on the 5′ fragments, whereas the A-type mismatch is strongly enriched in 3′ fragments (Figure 4A). Common guanosine or adenosine modification on tRNAs (Figure 4B) include m^1^G, m^2^G and m_2_^2^G on the 5’ half of tRNA or anticodon loop, and m^1^A on the T-loop or on the ninth position of specific tRNAs. The presence and relative abundance of these modifications on tRFs have not been extensively investigated, especially in bladder cancer.

High mismatch rate was detected by TGIRT-seq at specific positions on specific tRFs (patient #1 shown as example in Figures 4C,D). For example, G-type mismatch was detected at the ninth position on the highly abundant 5′ fragment from tRNA^Glu^ (Figure 4C), consistent with the known m^1^G site on the parental tRNAs. Interestingly, another highly abundant 5’ fragment, from tRNA^Gly^, does not have high mismatch rate detected, despite having guanosine at its ninth position. Across 12 tumor samples, the mismatch pattern at ninth position (Figure 4E) corresponds very well with previous measurements of mismatch on mature tRNAs: high mismatch rate on tRF^Asn^, tRF^Arg^, tRF^Gln^, tRF^Pro^ and tRF^iMet^, moderate mismatch rate on tRF^Glu^ and tRF^Thr^.

A-type mismatch at the ninth position was also detected on tRF^Asp^ (Figure 4C), corresponding to the reported m^1^A modification on tRNA^Asp^. Similarly, we detected G-type mismatch frequently at the 26th position on specific 5′ fragments across 12 samples (Figure 4F): high mismatch rate on tRF^Ile^, tRF^Leu^, tRF^Met^, tRF^Phe^, tRF^Ser^ and tRF^Trp^, moderate mismatch rate on tRF^Ala^, tRF^Asn^, tRF^Arg^ and tRF^Tyr^. Lastly, TGIRT detects overall high mismatch rate at m^1^A58 position on 3’ tRNA fragments across 12 samples with slightly lower rate on tRF^Ala^, tRF^Cys^, tRF^Glu^, tRF^Leu^ and tRF^Thr^ (Figure 4G) and very low mismatch on tRF^Asp^ (Figures 4D, G). Overall TGIRT-seq captures mismatch at specific positions on tRFs, with a mismatch pattern similar to that expected from the mismatch pattern of the corresponding tRNAs. This suggests modifications like m^1^G, m_2_^2^G and m^1^A are highly prevalent on tRFs.

### TGIRT-seq detects abundant rRFs with overall low mismatch rate but high mismatch at specific positions

**FIGURE 5:**
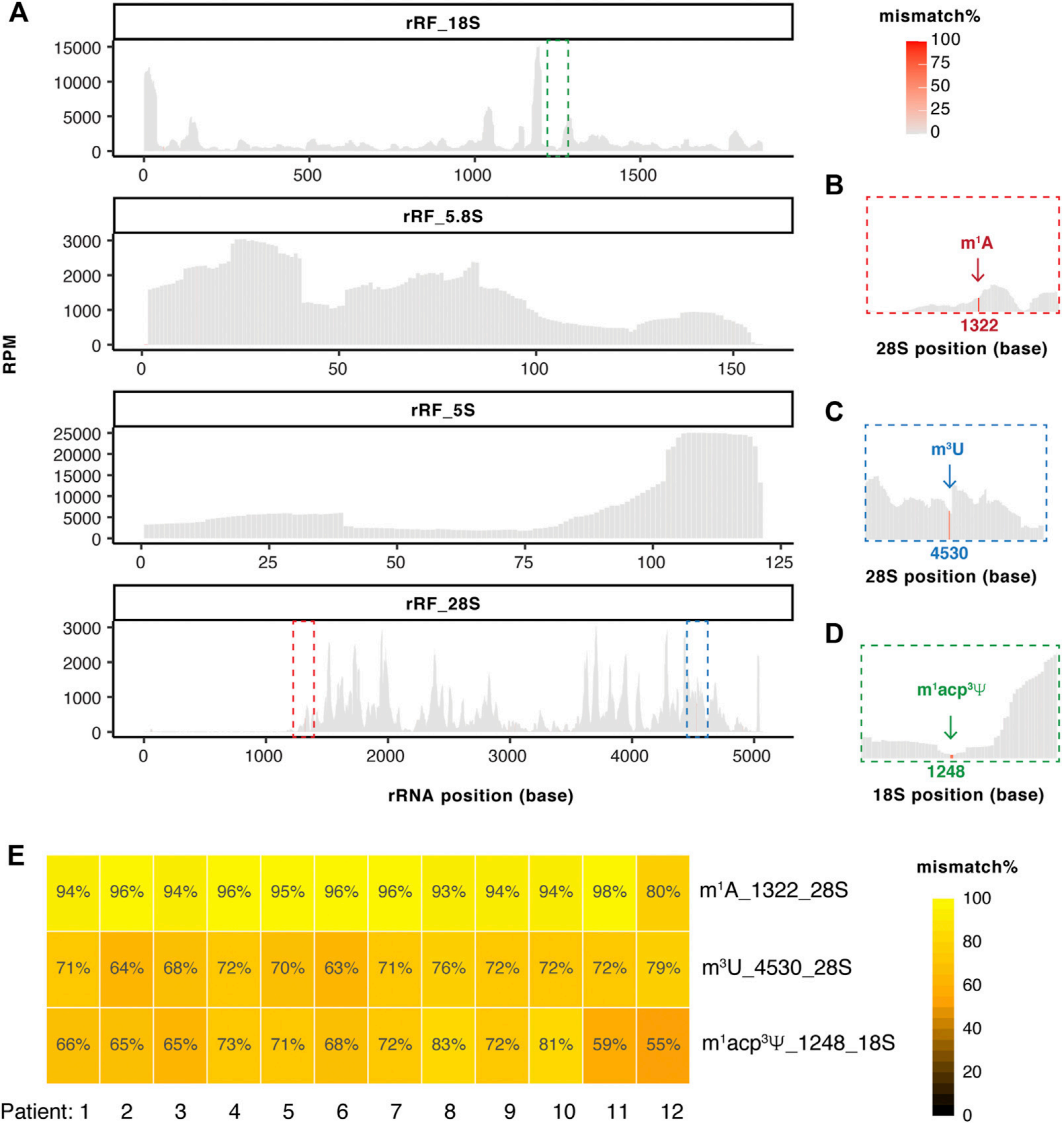
TGIRT-seq detects abundant rRFs with overall low mismatch rate and high mismatch at specific positions. **(A)** Example coverage plot of rRFs mapped on each rRNA sequences with mismatch highlighted at each position (patient #2 shown as example). Coverage plots for all 12 samples are shown in Supplementary Figures S5–8. Squared boxes are further zoomed in panel **(B)** to show high mismatch positions. **(B–D)** High mismatch at **(B)** position 1322 on 28S rRNA **(C)** position 4530 on 28SrRNA and **(D)** position 1248 on 18S rRNA are highlighted. **(E)** Patient-to-Patient variation of the mismatch% at the three high mismatch positions on rRFs **(B–D)**.

Another group of abundant nmsRNAs is rRFs (Figure 1). The rRF reads are mapped to all four mature rRNA sequences, 18, 28, 5.8 and 5S. rRFs are highly abundant with comparable RPMs to abundant miRs or tRFs. The rRF coverage along the length of rRNAs is not evenly distributed, as would be expected if they were random degradation products, but interestingly concentrated at discrete regions (Figure 5A, patient #2 shown as an example, all 12 samples shown in Supplementary Figure S5). Consistent with the dominant peak at 39 nt of all rRFs (Figure 2C), these discrete regions show up as peaks of around 39 nt at various sites on the rRNAs (Figure 5A). The rRFs from 18S RNA have the two highest peaks at 0, 1200 bases along the length or the RNA, and this general pattern is seen across 12 patients, with new rRF source sites towards 3’ end seen in patient #6 (Supplementary Figure S5). We do not know the explanation for the different pattern in individuals, but there may be interesting biological differences in the tumor accounting for the difference. Similar analysis was done for rRFs from 5.8S (Supplementary Figure S6), 5S (Supplementary Figure S7) and 28S (Supplementary Figure S8), which shows generally conserved patterns across 12 patients.

Unlike tRFs, rRFs are not associated with high mismatch reads on an overall view (Figure 5). However, mismatch is observed at specific position, for example position 1322 on fragments from 28S rRNA (Figure 5B). This position is known to bear m^1^A but has not been reported on the rRFs. We were able to detect high mismatch at position 4530 of 28S rRF (Figure 5C) corresponding to known m^3^U modification and position 1248 of 18S rRF (Figure 5D) corresponding to m^1^acp^3^Ψ. All these modifications are known to disrupt base pairing therefore induce mismatch during reverse transcription. We didn’t observe high mismatch (>10%) at positions consistent in multiple patients from 5.8S to 5S rRFs (Supplementary Figures S6, 7). Lastly, these three rRF mismatch sites are consistently detected among 12 patients with overall very high mismatch rate (Figure 5E). This suggests specific rRFs could harbor modifications from the parental rRNAs, which will require further future investigation.

### Specific and abundant YRNA fragments with overall low mismatch rate

**FIGURE 6:**
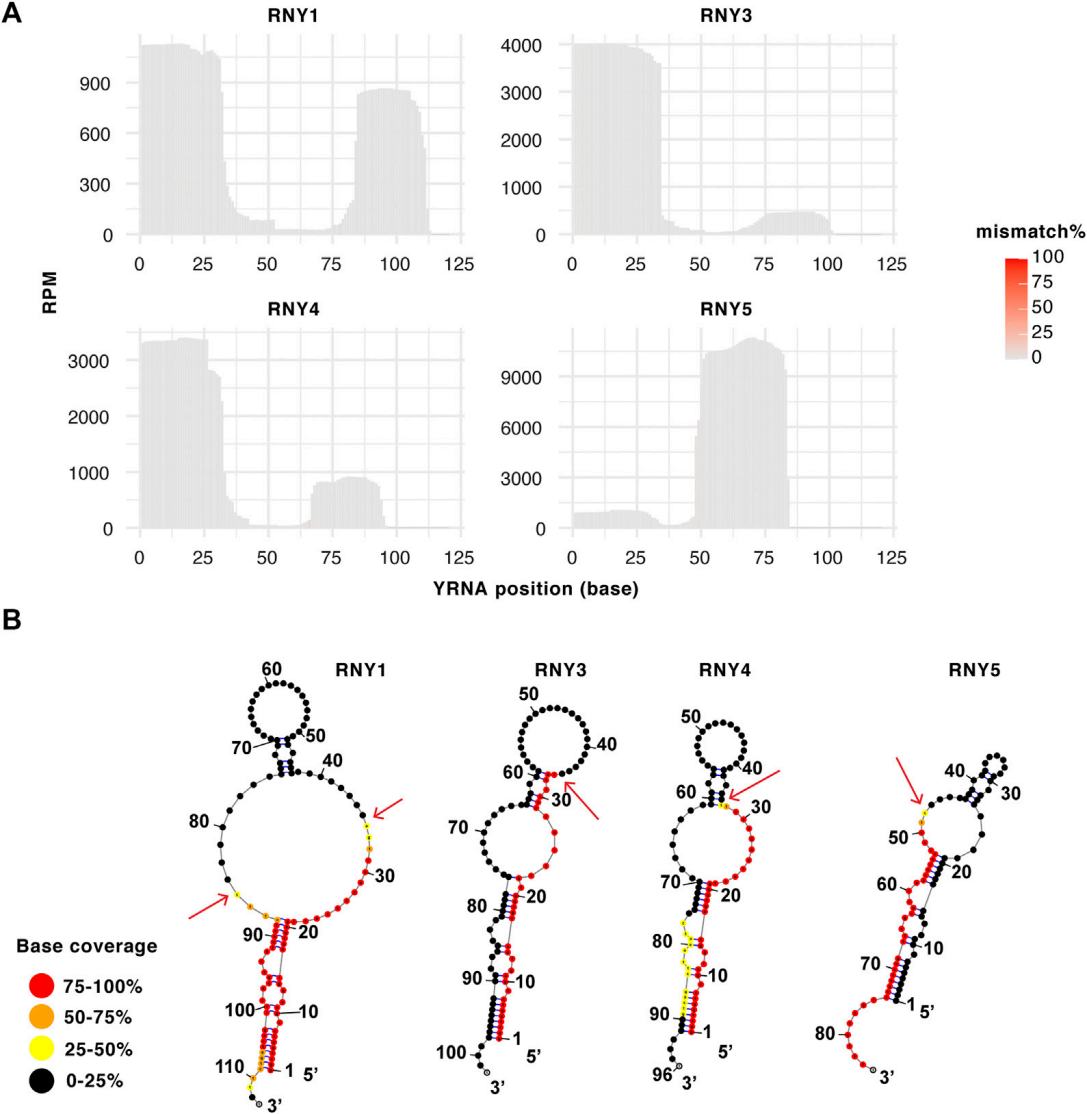
Specific Y-RNA fragments with overall low mismatch rate. **(A)** Example coverage plot of YRFs mapped on each Y-RNA sequences with mismatch highlighted at each position (patient #1 shown as example). Coverage plots for all 12 samples are shown in Supplementary Figure S9. **(B)** YRF base coverage shown with Y-RNA secondary structures. Base coverage is color coded with red showing highest coverage and black showing lowest coverage. YRF cleavage site as indicated by the coverage drop is indicated by arrow.

In addition to tRFs and rRFs, we also detected abundant YRNA fragments (YRF) from all four YRNAs, RNY1, RNY3, RNY4 and RNY5 (Figure 6A, patient #1 shown as an example, all 12 patient samples shown in Supplementary Figure S9). The most abundant YRF is 3′ fragment from RNY5 (∼10,000 RPM), which is close to the most abundant microRNA level (Figure 3B). Interestingly, the fragmentation pattern is very specific and generates 5′ and 3′ molecules similar in length to the tRNA halves, although they are not themselves exactly half of a YRNA (Figure 6B). YRNAs share conserved secondary structure with a ∼20 bp stem formed by annealing of the 5′ and 3’ ends, which is adjacent to a loop (preterminal loop) (Figure 6B). The YRF cleavage occurs at single-stranded region of YRNAs, especially within the preterminal loop of RNY1, 4 and 5 (Figure 6B). This cleavage pattern is highly consistent among 12 patient samples with some patient-to-patient variation in abundance (Supplementary Figure S9), suggesting specific cleavage. Overall, YRFs are not associated with high mismatch reads (Figure 6A).

### NmsRNAs in the 18–24 base range, that may enter argonaute complexes

Given the emerging reports of tRF involvement in Argonaute-mediated gene silencing activity (Maute et al., 2013; Kuscu et al., 2018; Ren et al., 2019), it is important to determine how many and which nmsRNAs are likely to enter Argonaute and potentially affect gene expression. We used the following criteria: 1) 18–24 base long and so expected to enter Argonaute complexes, 2) consistently detected in at least 10 out of the 12 samples, 3) present at an abundance comparable to that of microRNAs. As listed in Supplementary Table S1, 12 unique nmsRNA sequences in this size range were seen at an abundance of 500–15,000 RPM, an abundance at which we see 96 unique microRNA sequences (isomiRs were not combined due to sequence variations). These include tRF-1001, tRF-3001a, tRF-5027b and tRF-5004a (3 nt shorter than annotated sequence). All have been detected in AGO PAR-CLIP (photoactivatable ribonucleoside-enhanced crosslinking and immunoprecipitation) except tRF-1001 (Kumar et al., 2014; Kumar et al., 2015). The identities of nmsRNAs that have the potential to enter productively into Argonaute complexes by virtue of their length, and are present at an abundance comparable to that of microRNAs, is listed in Supplementary Table S1. Various rRFs have been detected associated with AGO (Guan and Grigoriev, 2021). However, the abundant rRFs that we have identified in this paper have not been reported in association with AGO, but this could either be because these rRFs are not sufficiently abundant in the cell lines where the AGO PAR-CLIP experiments were done, or because there is a mechanism that keeps these fragments away from being loaded into AGO. This analysis suggests that tRF-3001a, tRF-5027b and tRF-5004a should be studied further in BLCA for their microRNA-like activity, and the other nmsRNAs in Supplementary Table S1 may also emerge as being important for BLCA biology through mechanisms waiting to be elucidated.

## Discussion

We utilized TGIRT-seq of small RNAs that were size-selected to include RNAs that are usually discarded during microRNA profiling. The results identified a large array of non-microRNA-small RNAs (nmsRNAs) and associated modifications in bladder cancer tumor samples. nmsRNAs are as abundant as the well-studied microRNAs (Figure 1). nmsRNAs display different size distribution than microRNAs of 22 nucleotides, with a significant portion with a longer length (Figure 2). General abundance, cleavage patterns and potential modification sites were reported for nmsRNAs, including tRNA-derived fragments (tRFs), rRNA-derived fragments (rRFs) and YRNA-derived fragments (YRFs) (Figures 3–6). Overall, this highlights the usefulness of TGIRT-seq to profile both abundance and RNA modifications on small RNAs from clinical samples.

Emerging evidence suggests technical biases in small RNA-seq leads to under-representation of certain RNAs. The great abundance of nmsRNAs of length greater than 22 nucleotides (Figure 2) indicates they are often excluded by the size selection that is used during microRNA profiling. Furthermore, both internal RNA modifications that interfere with reverse transcription and terminal modifications that interfere with ligation could lead to under-cloning (Shi et al., 2021). Here we utilized TGIRT, a thermostable group-II intron reverse transcriptase based on bacterial retrotransposons, that has been developed into a powerful research tool (Belfort and Lambowitz, 2019). TGIRT can mitigate the RT-stalling problem caused by certain internal modifications, but RNAs with other modifications may still be under-cloned. Newer techniques to tackle this gap in true short RNA representation are needed (Alfonzo et al., 2021).

The most abundant nmsRNAs are tRFs and rRFs (Figures 3–5), both with sequences present at similar abundance as the most abundant microRNAs (500-15,000 RPM). Diverse biological functions of tRFs have been reported in cancers (Su et al., 2020b). The highly abundant tRFs detected in BLCA samples by this study include 19-nt tRF-1001 from tRNA^Ser^ (Figure 3 and Supplementary Table S1) that was initially reported in cancer cell lines and associated with cell proliferation (Lee et al., 2009). Another abundant tRF reported here is 18-nt tRF-3001a from tRNA^Leu^, which has been shown to enter Argonaute complexes (Kumar et al., 2014) and is capable of repressing target gene expression in a seed sequence match manner (Kuscu et al., 2018). In addition, both 5′ and 3′ tRNA halves from tRNA^Glu^, tRNA^Gly^, tRNA^Lys^ and tRNA^Val^ appear to be very abundant in BLCA samples (Figure 3). Despite their high abundance, the functions of these tRNA halves have not been extensively studied in cancers. tRNA halves can be induced by various stress conditions but can also be detected at endogenous non-stress condition. While 5′ tRNA halves have been associated with translational repression (Ivanov et al., 2011), non-coding RNA levels and histone levels (Boskovic et al., 2020) and more recently tRNA transcription (Chen et al., 2021), much less work has been done on 3’ tRNA halves. So far, correlational studies based on tRF expression in BLCA patients suggests tRFs very likely play a role in BLCA gene regulation (Telonis et al., 2019; Papadimitriou et al., 2020), however future investigation in a refined experimental system is required to establish a direct association.

In addition to tRFs, we also detected abundant rRFs and YRFs in BLCA samples (Figures 5, 6). Biological significance and functions are still awaiting investigation for rRFs and YRFs, as recent evidence suggests they are not random degradation products. Profiling of rRFs (<34 nt) in 1000 Genome Project revealed sex- and population-dependent patterns (Cherlin et al., 2020). Furthermore, rRFs ∼20 nt are identified associated with Argonaute and paired with cellular transcripts with enriched motifs that are different from microRNA rules (Guan and Grigoriev, 2021). Intriguingly, the abundant rRFs detected in this study were not identified in the Argonaute association studies. Similarly, YRFs ∼31 nt could be regulated by stress and were not found associated with Ago, as recently reviewed (Guglas et al., 2020). This could be either because these nmsRNAs are not abundant in the cell lines where the association studies were performed or have been under-cloned due to RT-stalling modifications, or more intriguingly, have other Ago-independent functions. Further investigation is needed to shed light on these abundant nmsRNAs, both the Ago-compatible species and the longer species. Interestingly, relative distribution among different RNA sub-groups is quite variable from sample to sample, with some samples having higher percentage of nmsRNAs than others (Figure 1). In the future, it will be worthwhile to survey potential causes for such difference in a more systematic analysis. Such causes could be technical (sample handling or contamination) or biological (dysregulation of small RNA homeostasis or correlation with certain clinical features).

We also tested whether TGIRT-mediated mismatches identify known modification sites (m^1^G, m^1^A and m_2_^2^G) on tRFs and rRFs (Figure 4 and Figure 5). In general, the mismatch pattern on tRFs corresponds very well with the sites of modification detected previously on mature tRNAs (Clark et al., 2016; Behrens et al., 2021). The very low m^1^A mismatch on tRF^Asp^ (Figures 4D,G) is consistent with the very low m^1^A58 on tRNA^Asp^. Similarly, the A1322 position is known to bear m^1^A on large ribosomal subunit RNA across species and is catalyzed by nucleomethylin (also known as RRP8) in humans (Waku et al., 2016; Sharma et al., 2018). The U1248 position of 18S is known to be m^1^acp^3^Ψ modified and is located within the ribosome decoding region (Meyer et al., 2011). The U4530 position of 28S is known to be m^3^U modified (Tan et al., 2021). These three rRF modification sites were detected with high mismatch by TGIRT (Figure 5). Interestingly, 18S:1248 (m^1^acp^3^Ψ) was suggested to have a lower modification level based on mismatch pattern from long RNA-seq in TCGA tumors, especially READ, UCEC and COAD (Tan et al., 2021). Surprisingly, although rRNA modifications on human ribosomes have very recently been visualized by Cryo-EM (Natchiar et al., 2017), a lot of the rRNA modification enzymatic processes are not well elucidated in humans. The mismatch profile may also be used to identify unannotated modification sites in the parental RNAs in the future but will need to be verified with orthogonal methods. How could these modifications alter in disease conditions and whether they have any impact on ncRNA functions will be an interesting prospective research topic. Recently we reported m^1^A impedes tRF-3 gene-silencing activity and is over-expressed in BLCA tumor, coinciding with over-expression of the writer enzyme proteins TRMT6/61A and dysregulation of the tRF-3 targetome (Su et al., 2022). In addition to bladder cancer, TRMT6/61A is also over-expressed in liver cancer and glioma. This is particularly important for cancer since disruption of many RNA modification enzymes has been linked to cancer (Janin et al., 2020; Chujo and Tomizawa, 2021).

Alterations in urinary RNA modification levels hold potential to serve as a non-invasive way to diagnose patients with BLCA and moreover as a monitoring tool to detect disease recurrence. Several studies have reported elevated levels of modified nucleosides detected in urine from BLCA patients, including m^1^A (Kvist et al., 1993; Zhang et al., 2014; Sun et al., 2015). The significance of miRNA in BLCA carcinogenesis and as urine cancer biomarkers has been well studied (Yoshino et al., 2013; Hammouz et al., 2021). However, the role of nmsRNAs in BLCA pathogenesis, or as clinically relevant biomarkers, is only beginning to emerge. Interestingly, urine was one of the biofluids with the highest proportion of tRFs detected in healthy donors (Yeri et al., 2017; El-Mogy et al., 2018; Godoy et al., 2018). Both YRNAs and YRFs are recognized as biomarkers in various malignancies as reviewed (Guglas et al., 2020). They are generally downregulated in BLCA and a low expression of RNY1, 3 and 4 is associated with muscle invasiveness, lymph node metastases, advanced stage, and an unfavorable prognosis (Tolkach et al., 2017). Our observed specific YRNA cleavage pattern taken together with the previous knowledge of YRNA in BLCA suggests a potential regulatory role in the pathogenesis. Yet, whether YRNAs or YRFs are useful urine biomarkers require further investigation.

## Data Availability

The data analyzed in this study is subject to the following licenses/restrictions: The raw sequencing data for small RNA TGIRT-seq in BLCA patients are protected by European and national regulations regarding data privacy laws but will be available upon request. Requests should be directed to Rune Ougland (runoug@vestreviken.no) and will be forwarded to the Data Protection Officer and the Ethics Committee for legal- and ethical evaluation. The data will be available for 10 years after publication and if the requesting institution has implemented the European GDPR, or is able to sign the Standard Contractual Clauses for international transfers, the process will take <6 weeks. Otherwise, inter-institutional negotiation is necessary which may prolong the wait time. Meanwhile, we provide the gene counts as Supplementary Data.
