# Supplementary material for: Characterization of novel small non-coding RNAs and their modifications in bladder cancer using an updated small RNA-seq workflow
Source: Front Mol Biosci. 2022 Jul 18;9:887686. doi: 10.3389/fmolb.2022.887686 (PMC9340255; doi:10.3389/fmolb.2022.887686)
Supplement: Supplementary file 1 [file DataSheet1.PDF]

Supplementary Information for

Characterization of novel small non-coding RNAs and their modifications in bladder cancer using an updated small RNA-seq workflow

Supplementary Figures

Supplementary Figure 1. Mapping strategy for miRs and non-miRs.

Supplementary Figure 2. microRNAs and non-microRNA small RNAs show distinct size distribution.

Supplementary Figure 3. Size distribution of tRF subtypes.

Supplementary Figure 4. TGIRT-seq captures mismatch at specific positions corresponding to RNA modification sites.

Supplementary Figure 5. TGIRT-seq detects abundant 18S rRFs with overall low mismatch rate.

Supplementary Figure 6. TGIRT-seq detects abundant 5.8S rRFs with overall low mismatch rate.

Supplementary Figure 7. TGIRT-seq detects abundant 5S rRFs with overall low mismatch rate.

Supplementary Figure 8. TGIRT-seq detects abundant 28S rRFs with overall low mismatch rate.

Supplementary Figure 9. Specific Y-RNA fragments with overall low mismatch rate.

Supplementary Table

Supplementary Table 1. Abundant nmsRNA sequences detected in BLCA tumor samples

Supplementary Figures

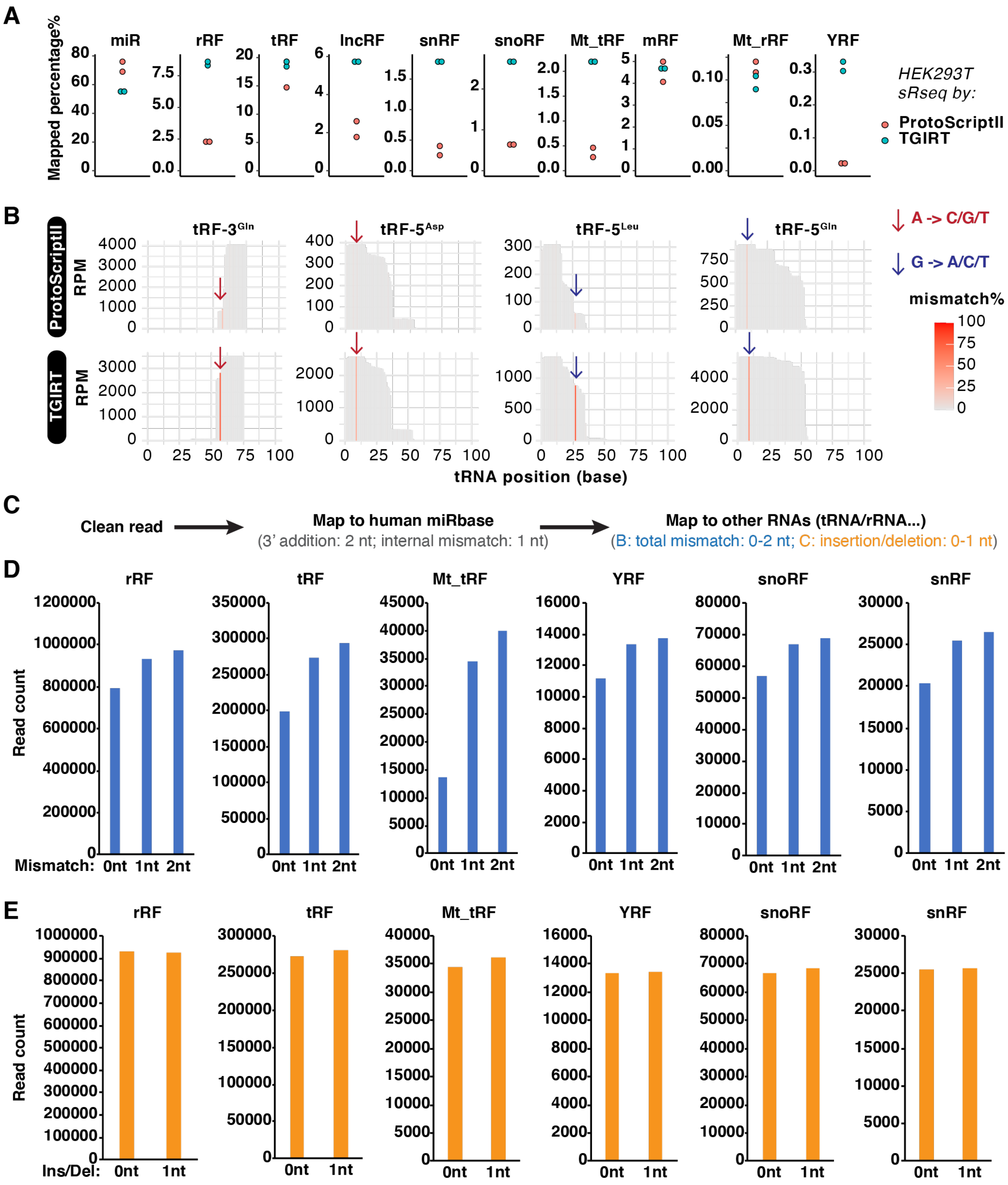

Supplementary Figure 1. Mapping strategy for miRs and non-miRs.

(A-B) Side-by-side comparison of small RNA-seq done by ProtoScriptII and TGIRT of the same HEK293T RNAs. (A) Mapping percentage of different small RNA species by ProtoScriptII (n = 2) and TGIRT (n = 2). (B) Y-axis represents Reads per Million mapped reads. Mismatch position

is highlighted by colored arrows (red arrows for A-type mismatch and blue arrows for G-type mismatch).

(C) Scheme of mapping strategy. Clean reads are first mapped to miRs (allowing 2 nucleotides 3' addition and 1 nucleotide internal mismatch), and remaining reads mapped to non-miRs (allowing 0-2 nucleotides total mismatch and 0-1 nucleotide insertion/deletion).

(D) Testing different tolerance in mismatch for non-miRs mapping. Total mismatch of 0-2 nucleotides were tested, allowing mismatch increased the mapping rate.

(E) Testing different tolerance in internal insertion or deletion for non-miR mapping. Allowing 1 nucleotide insertion/deletion did not improve the mapping rate.

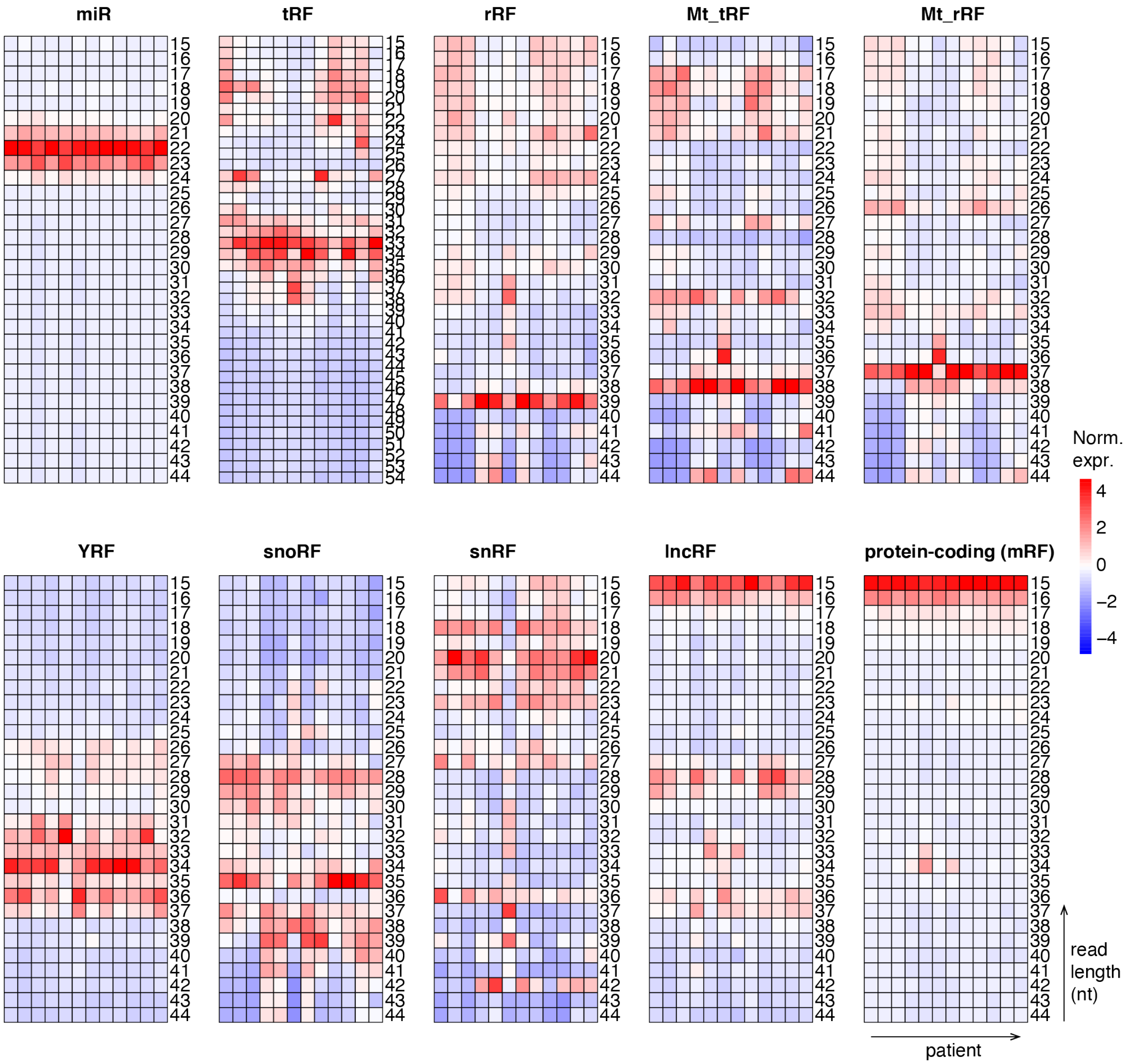

Supplementary Figure 2. microRNAs and non-microRNA small RNAs show distinct size distribution.

Length distribution of each small RNA subtypes shown as heatmap. Each column represents one patient sample (n = 12), and color represents normalized expression in each patient (red = high expression, blue = low expression).

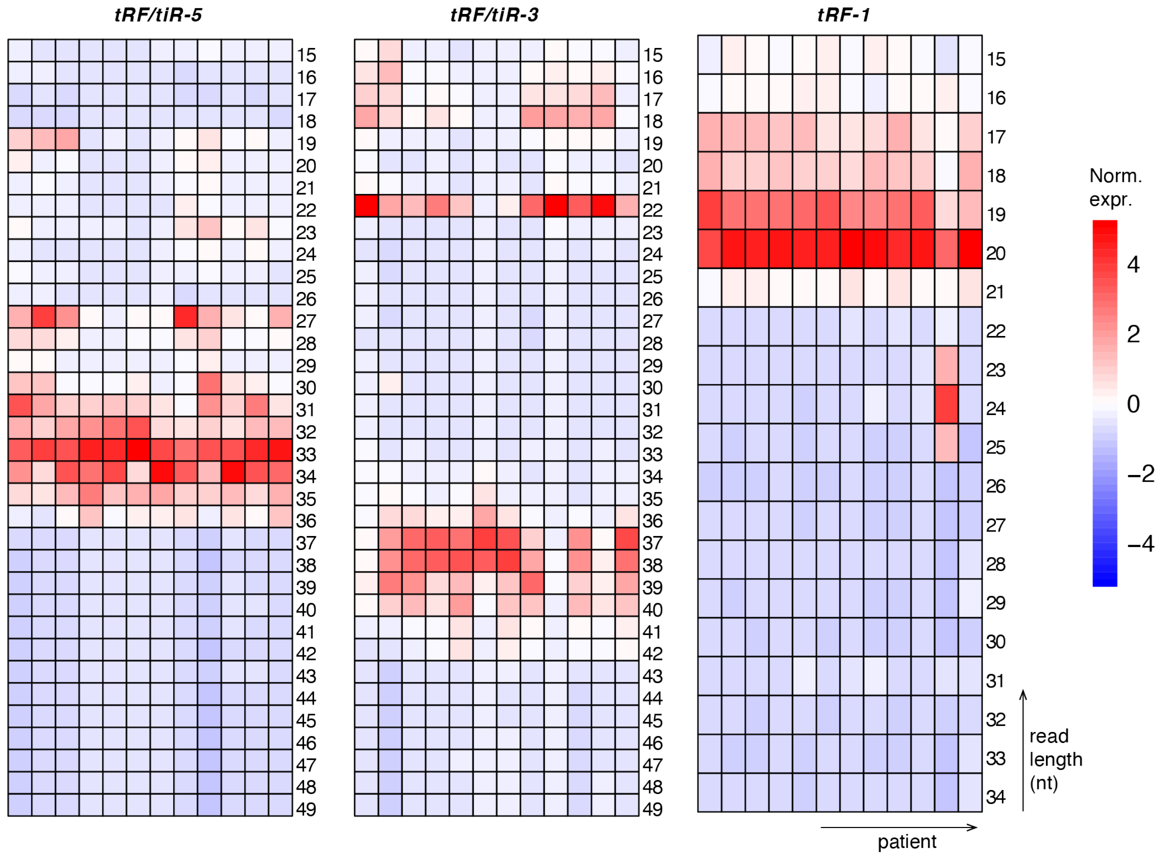

Supplementary Figure 3. Size distribution of tRF subtypes.

Length distribution of each tRF subtypes shown as heatmap. Each column represents one patient sample (n = 12), and color represents normalized expression in each patient (red = high expression, blue = low expression).

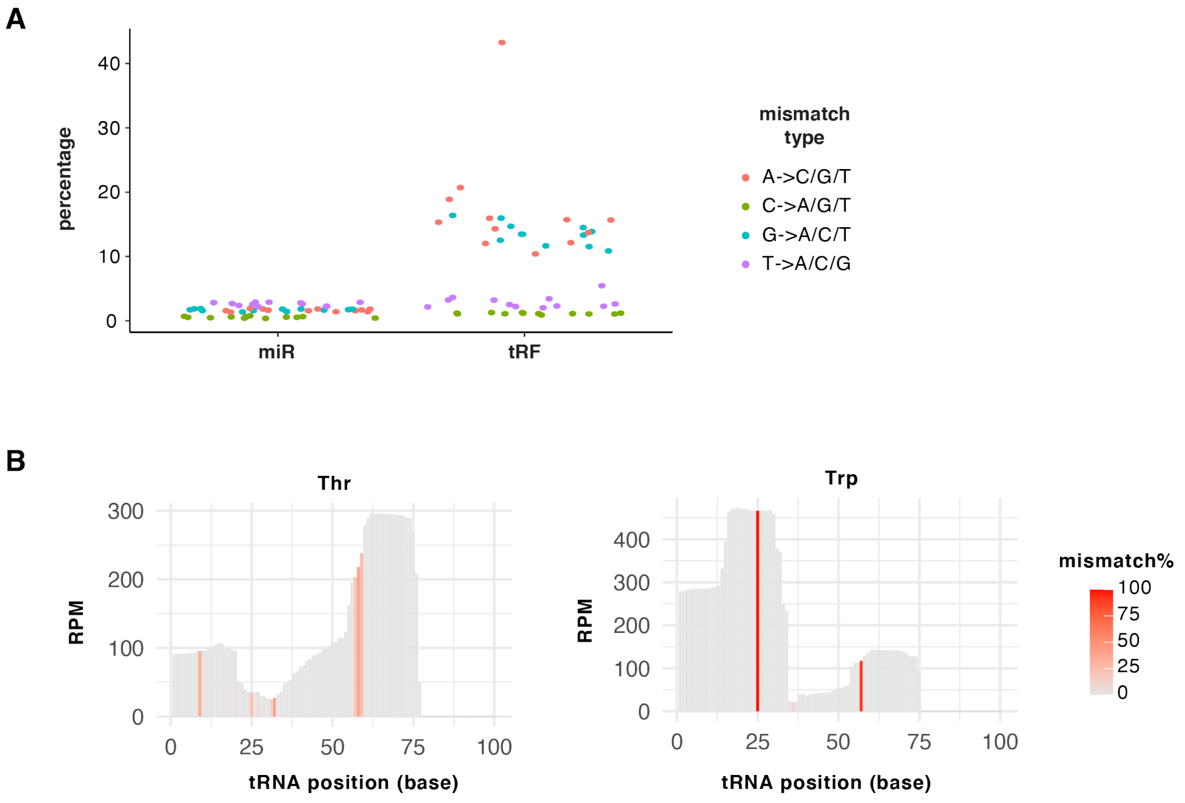

Supplementary Figure 4. TGIRT-seq captures mismatch at specific positions corresponding to RNA modification sites.

(A) A- and G- type mismatch is abundantly detected in tRFs by TGIRT-seq, compared to miRs. Each dot represents one patient sample (n = 12), separated by mismatch types (by color) and tRF types (X-axis). Y axis represents the percentage of reads that contain specific type of mismatch. (B) Example coverage plot for tRF^{Thr} and tRF^{Trp} with mismatch rate highlighted at each position in one sample.

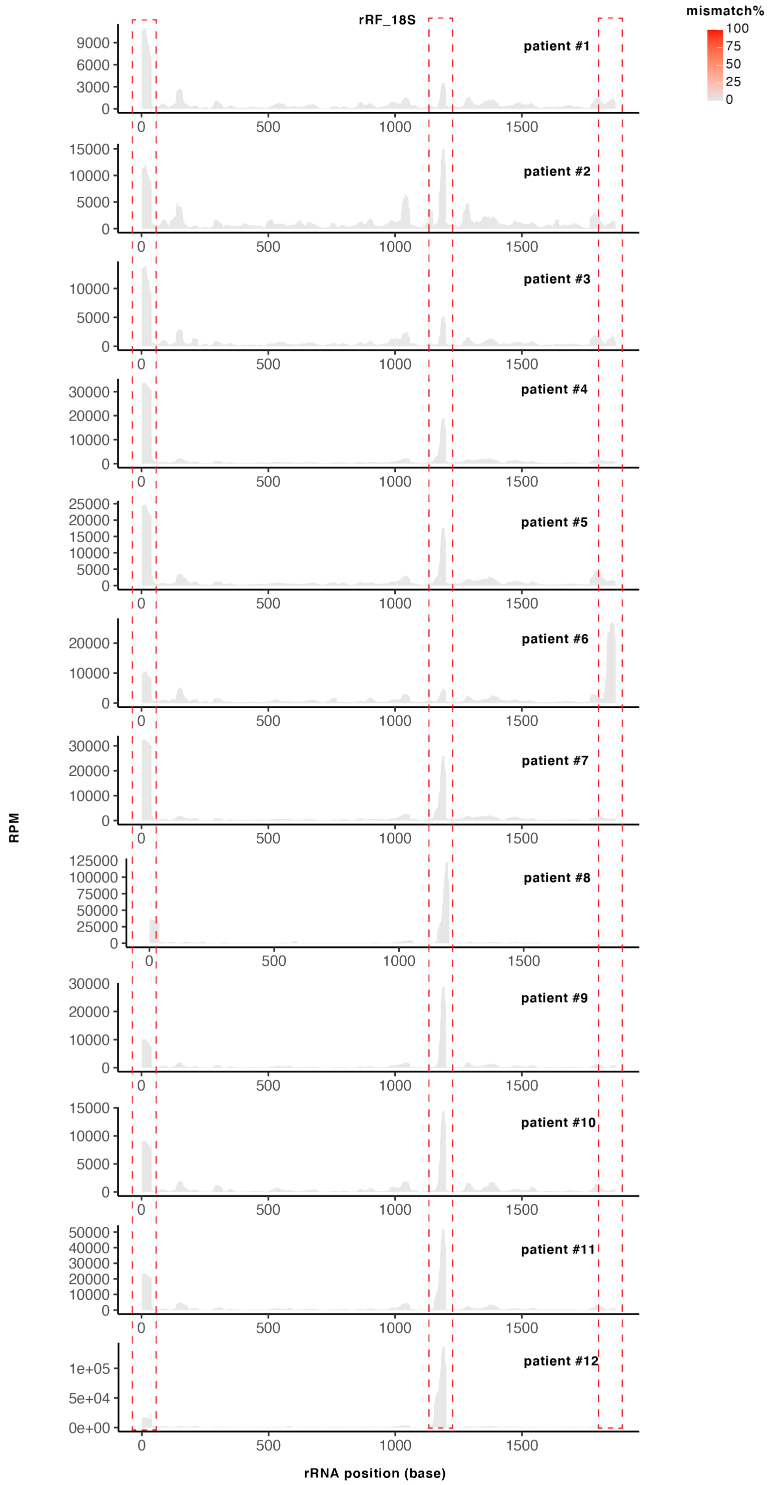

Supplementary Figure 5. TGIRT-seq detects abundant 18S rRFs with overall low mismatch rate.

rRFs mapped to 18S rRNA are plotted for 12 tumor samples.

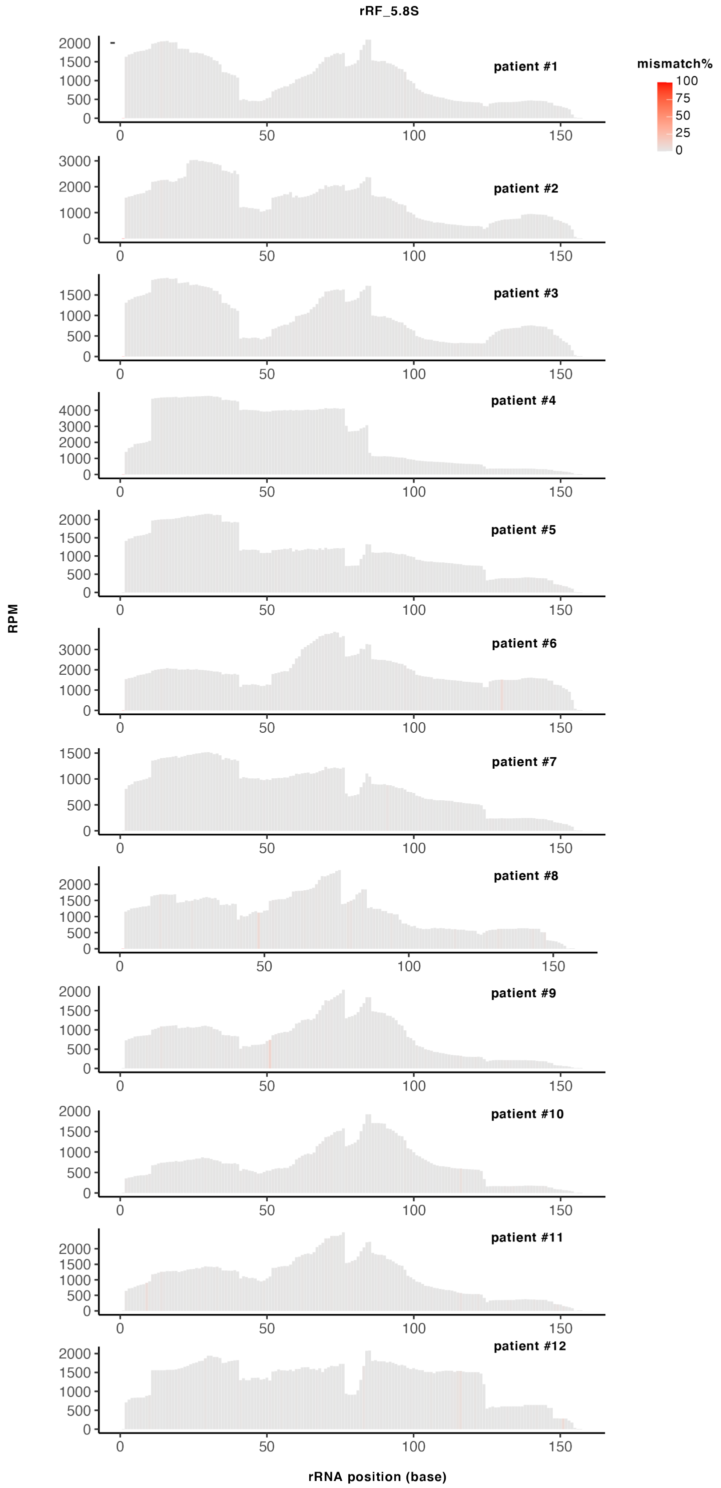

Supplementary Figure 6. TGIRT-seq detects abundant 5.8S rRFs with overall low mismatch rate.

rRFs mapped to 5.8S rRNA are plotted for 12 tumor samples.

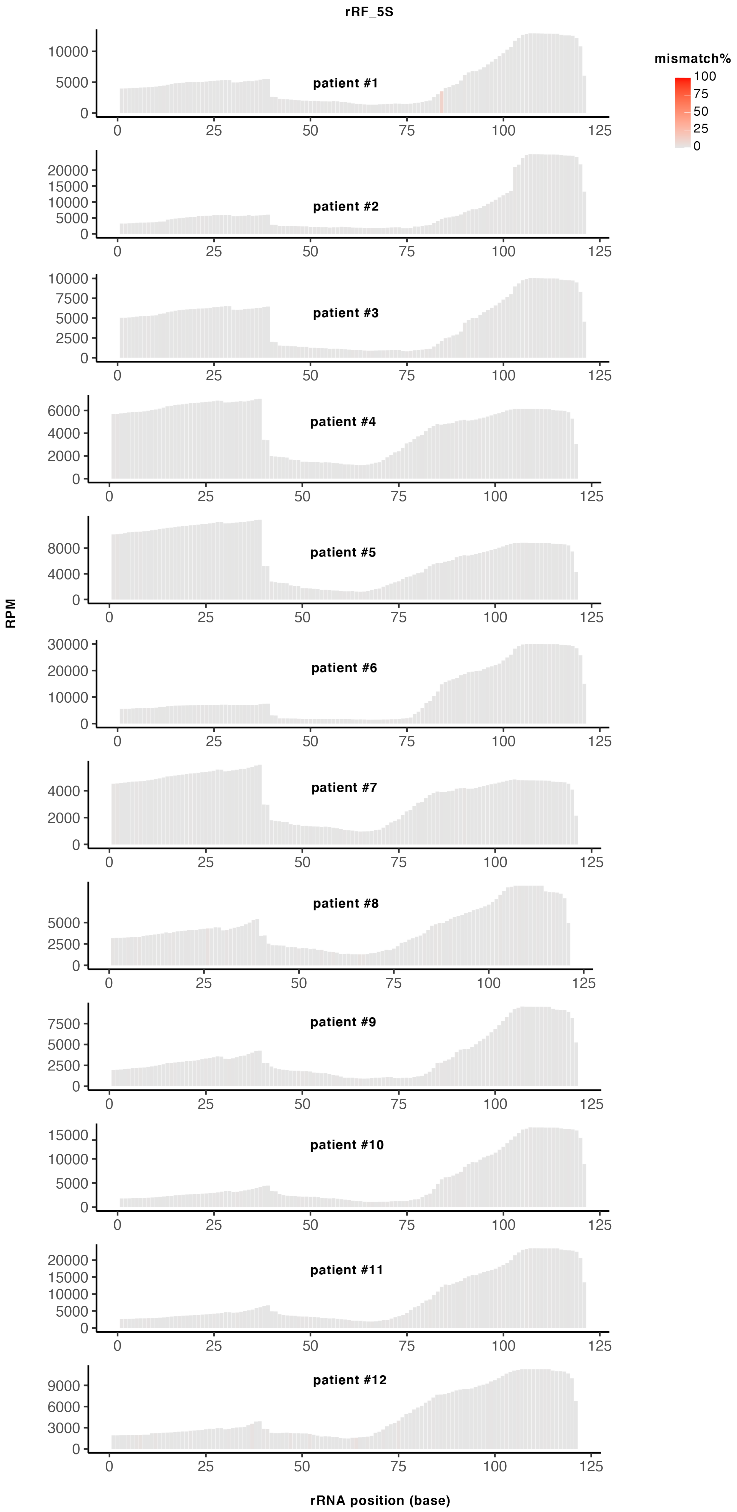

Supplementary Figure 7. TGIRT-seq detects abundant 5S rRFs with overall low mismatch rate.

rRFs mapped to 5S rRNA are plotted for 12 tumor samples.

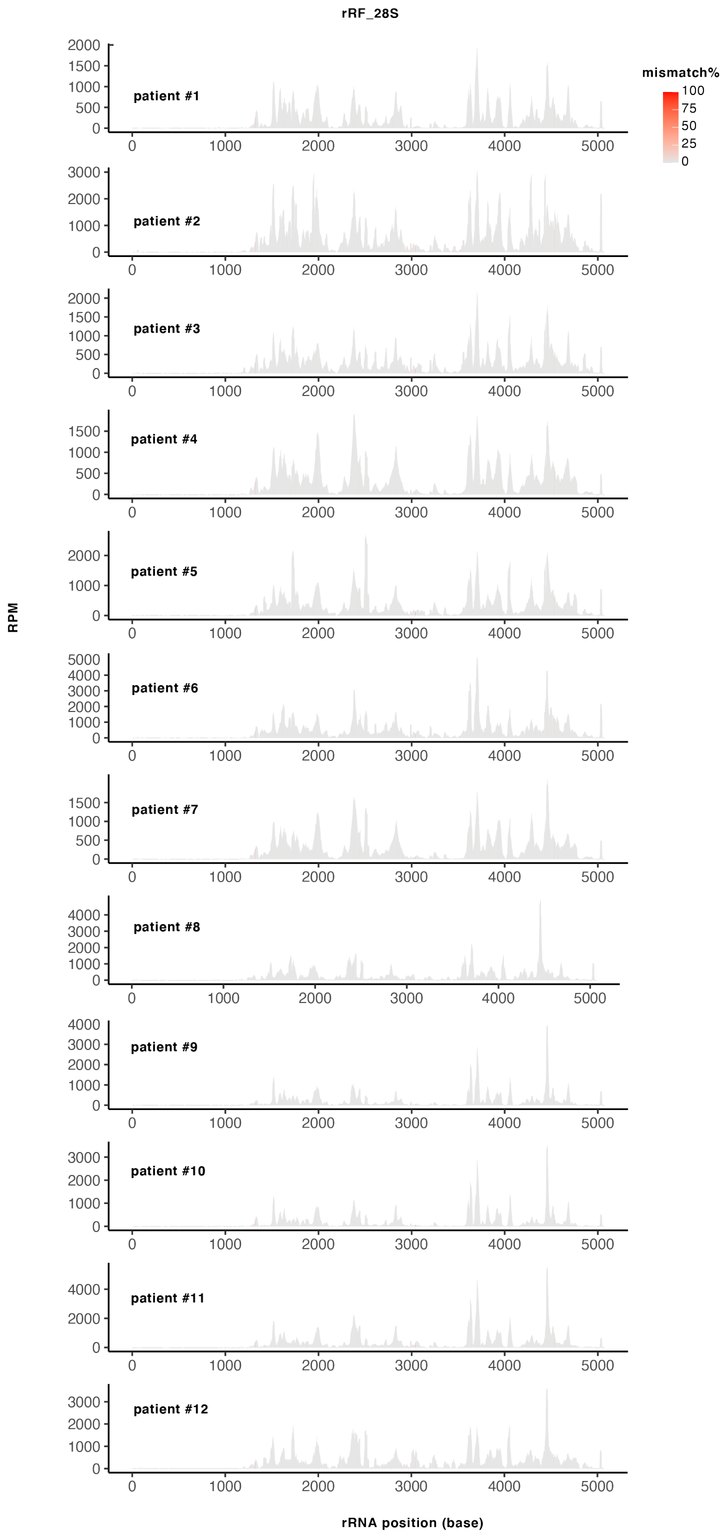

Supplementary Figure 8. TGIRT-seq detects abundant 28S rRFs with overall low mismatch rate.

rRFs mapped to 28S rRNA are plotted for 12 tumor samples.

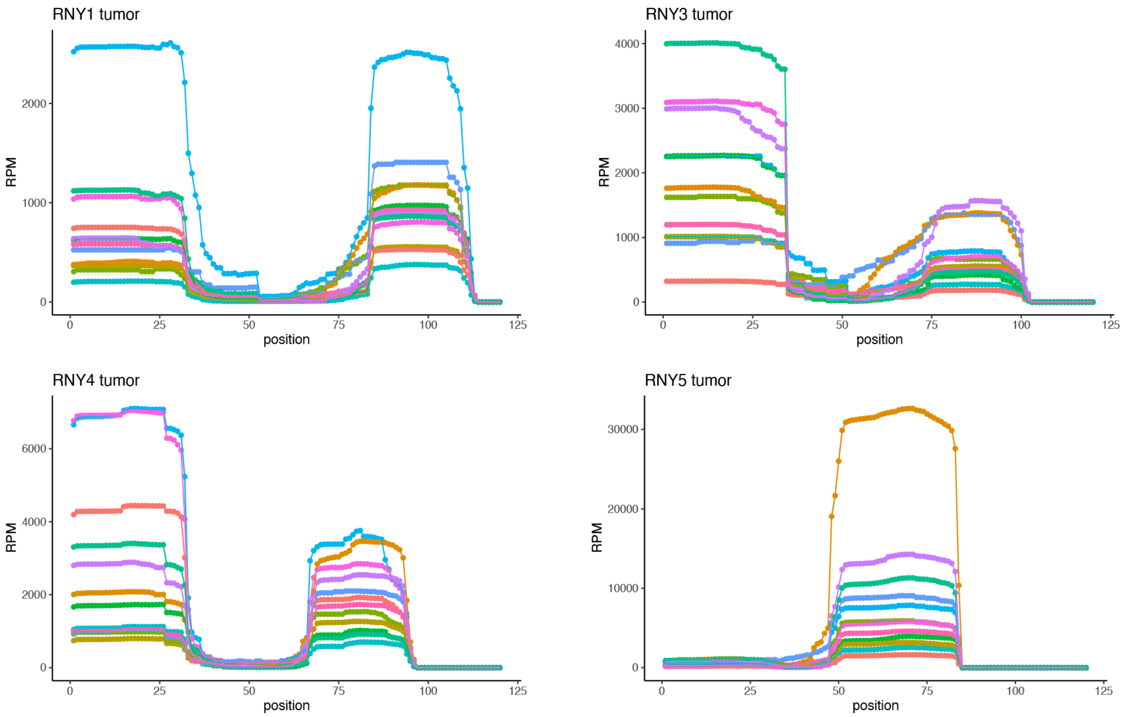

Supplementary Figure 9. Specific Y-RNA fragments with overall low mismatch rate.

Overlay of coverage plots of YRFs mapped on each Y-RNA sequences with each color representing one sample (n = 12).

Supplementary Table 1. Abundant nmsRNA sequences detected in BLCA tumor samples

Origin	Length	Sequence	Mean abundance (RPM)	Detected in AGO PARCLIP
tRF-1001 from tRNA-Ser-TGA-1-1 (1-19)	19	GAAGCGGGTGCTCTTATTT	2758.75169	No
tRF-1001 from tRNA-Ser-TGA-1-1 (1-20)	20	GAAGCGGGTGCTCTTATTTT	4877.07275	No
tRF-3001a from tRNA-Leu-AAG-4-1 (68-85)	18	ATCCCACCGCTGCCACCA	1252.98735	Yes
5p-tRF from tRNA-Gly-GCC-2-3 (1-19) *shorter than tRF-5004a	19	GCATTGGTGGTTCAGTGGT (also map to non-tRNA region)	2110.2088	Yes
tRF-5027b from tRNA-Val-CAC-1-1 (1-23)	23	GTTTCCGTAGTGTAGTGGTTATC (also map to non-tRNA region)	1043.73918	Yes
rRF (18S 1-18)	18	TACCTGGTTGATCCTGCC	1013.34257	No
rRF (18S 1-21)	21	TACCTGGTTGATCCTGCCAGT	1033.04123	No
rRF (18S 1-24)	24	TACCTGGTTGATCCTGCCAGTAGC	925.977612	No
rRF (18S 1181-1201)	21	AAAGCTGAAACTTAAAGGAAT	5486.60967	No
rRF (18S 1180-1201)	22	CAAAGCTGAAACTTAAAGGAAT	3040.28312	No
rRF (18S 1178-1201)	24	TGCAAAGCTGAAACTTAAAGGAAT	6492.04717	No
rRF (45S pre-rRNA 6596-6614)	19	TCGTACGACTCTTAGCGGT	3692.38967	No
